# Supplementary material for: A Printable Device for Measuring Clarity and Colour in Lake and Nearshore Waters
Source: Sensors (Basel). 2019 Feb 22;19(4):936. doi: 10.3390/s19040936 (PMC6413171; doi:10.3390/s19040936)
Supplement: Supplementary file 1 [file sensors-19-00936-s001.zip › Supplementary_data/Supplementary-data-1/Supplementary-data-1-weight.PDF]

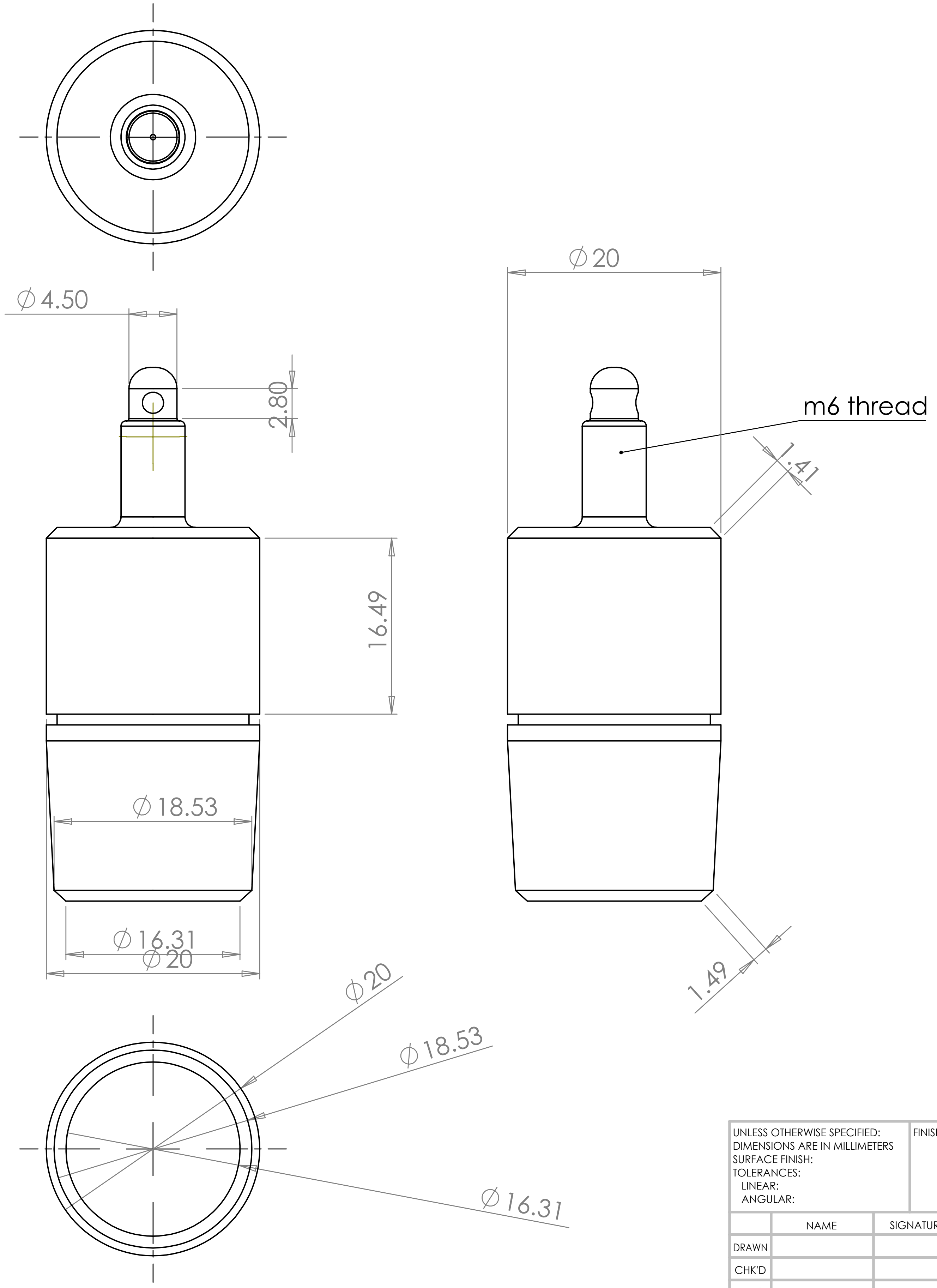

UNLESS OTHERWISE SPECIFIED:<br>DIMENSIONS ARE IN MILLIMETERS<br>SURFACE FINISH:<br>TOLERANCES:<br>LINEAR:<br>ANGULAR:	FINISH:	DEBURR AND BREAK SHARP EDGES	DO NOT SCALE DRAWING	REVISION	

	NAME	SIGNATURE	DATE		TITLE:
DRAWN					
CHK'D					
APPV'D					
MFG					
Q.A				MATERIAL:	DWG NO. weight — A3
				WEIGHT:	SCALE:2:1 — SHEET 1 OF 1
