# Supplementary material for: A Printable Device for Measuring Clarity and Colour in Lake and Nearshore Waters
Source: Sensors (Basel). 2019 Feb 22;19(4):936. doi: 10.3390/s19040936 (PMC6413171; doi:10.3390/s19040936)
Supplement: Supplementary file 1 [file sensors-19-00936-s001.zip › Supplementary_data/Supplementary-data-5/Supplementary-data-5-finger-strap.PDF]

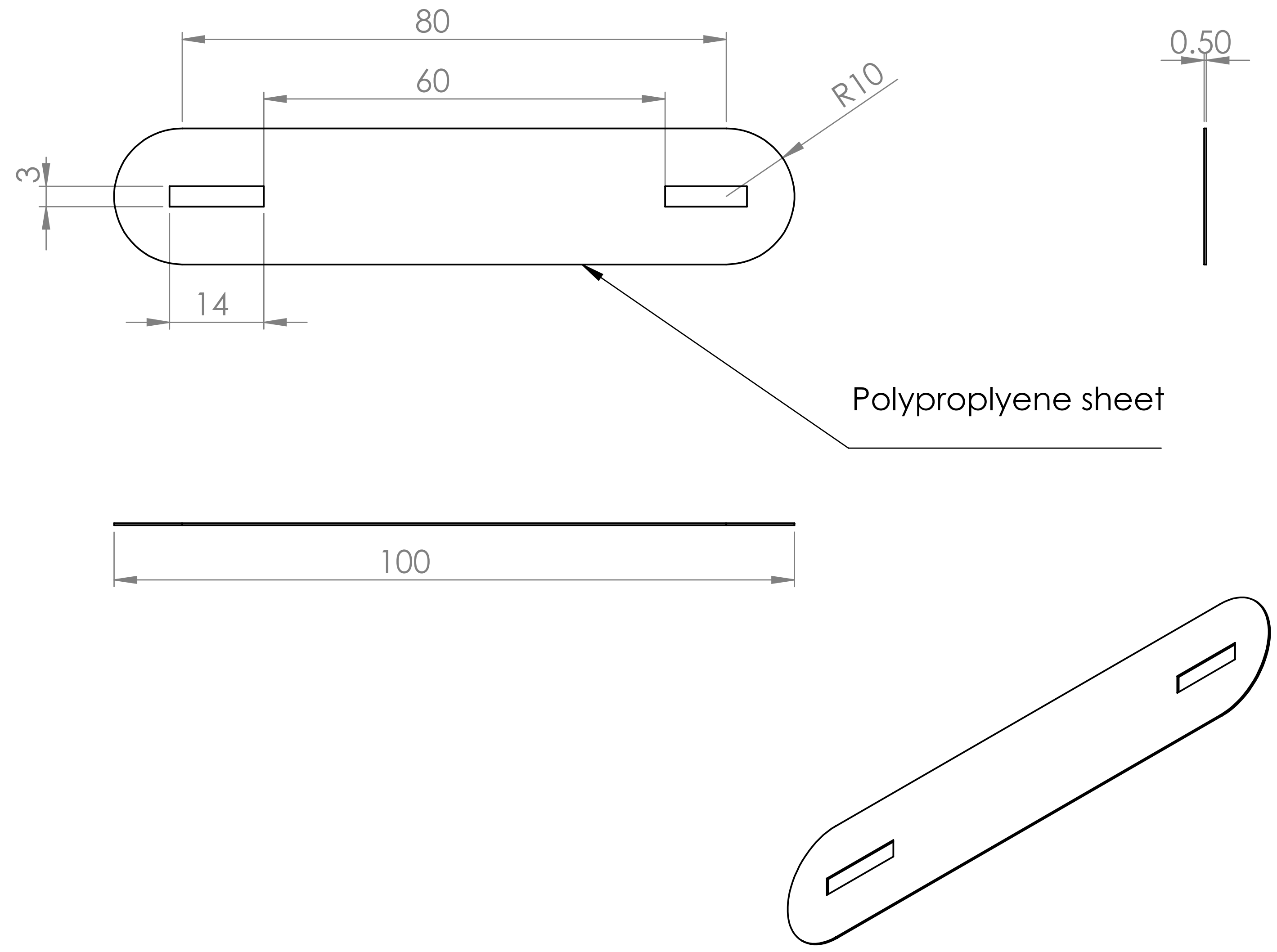

UNLESS OTHERWISE SPECIFIED: DIMENSIONS ARE IN MILLIMETERS SURFACE FINISH: TOLERANCES: LINEAR: ANGULAR:	FINISH:	DEBURR AND BREAK SHARP EDGES	DO NOT SCALE DRAWING	REVISION

	NAME	SIGNATURE	DATE
DRAWN			
CHK'D			
APPV'D			
MFG			
Q.A			

TITLE:

Polyproplyene strap as manufactured

MATERIAL:

WEIGHT:

DWG NO. strap

A3

SCALE:1:1

SHEET 1 OF 1

SOLIDWORKS Educational Product. For Instructional Use Only.
