# Supplementary material for: A Printable Device for Measuring Clarity and Colour in Lake and Nearshore Waters
Source: Sensors (Basel). 2019 Feb 22;19(4):936. doi: 10.3390/s19040936 (PMC6413171; doi:10.3390/s19040936)
Supplement: Supplementary file 1 [file sensors-19-00936-s001.zip › Supplementary_data/Supplementary_data_read_me.pdf]

Supplementary Data: A Printable Device for Measuring Clarity and Colour in Lake and Nearshore Waters

Robert J.W. Brewin[1,2,†,*], Thomas G. Brewin[3,†], Joseph Phillips[3,4], Sophie Rose[3,4], Anas Abdulaziz[5], Werenfrid Wimmer[6], Shubha Sathyendranath[1,2] and Trevor Platt[1]

[1] Plymouth Marine Laboratory, Plymouth, Devon, PL1 3DH, UK
[2] National Centre for Earth Observation, Plymouth Marine Laboratory, Plymouth, Devon, PL1 3DH, UK
[3] Chatham and Clarendon Grammar School, Ramsgate, Kent, CT11 9BB, UK
[4] Faculty of Science and Technology, Bournemouth University, Bournemouth, Dorset, BH12 5BB, UK
[5] CSIR-National Institute of Oceanography, Regional Centre Kochi, Kerala, 682018, India
[6] Ocean and Earth Science, National Oceanography Centre Southampton, University of Southampton, Southampton, Hampshire, SO14 3ZH, UK

† These authors contributed equally to this work
*Correspondence: robr@pml.ac.uk; Tel.: +44-1752-633100

The following files are provided as supplementary data to the article. These files are provided under 5 folders.

1) Supplementary-data-1
    This folder contains technical drawings of the two weights developed for the mini-Secchi device.
    - Supplementary-data-1-weight.PDF
    - Supplementary-data-1-weight-with-attachment.PDF
2) Supplementary-data-2
    This folder contains STL files of the two weights developed for the mini-Secchi device.
    - Supplementary-data-1-weight.STL
    - Supplementary-data-1-weight-with-attachment.STL
3) Supplementary-data-3
    This folder contains STL files of the casing, bobbin and handle for the mini-Secchi device.
    - Supplementary-data-3-bobbin-bottom.STL
    - Supplementary-data-3-bobbin-top.STL
    - Supplementary-data-3-casing-side-1.STL
    - Supplementary-data-3-casing-side-2.STL
    - Supplementary-data-3-handle.STL
    - Supplementary-data-3-handle-grip.STL
4) Supplementary-data-4
    This folder contains an example vinyl print file of the Forel Ule colour scale for the mini-Secchi device.
    - Supplementary-data-4-vinyl-print-colour-scale-example.pdf
5) Supplementary-data-5
    This folder contains a technical drawing of the finger strap for the mini-Secchi device.
    - Supplementary-data-5-finger-strap.PDF

These files are licensed under the Creative Commons Attribution-ShareAlike 4.0 International (CC BY-SA 4.0) License. To view a copy of the license, visit https://creativecommons.org/licenses/by-sa/4.0/.
