# Supplementary figures and images for: A Printable Device for Measuring Clarity and Colour in Lake and Nearshore Waters
Source: Sensors (Basel). 2019 Feb 22;19(4):936. doi: 10.3390/s19040936 (PMC6413171; doi:10.3390/s19040936)

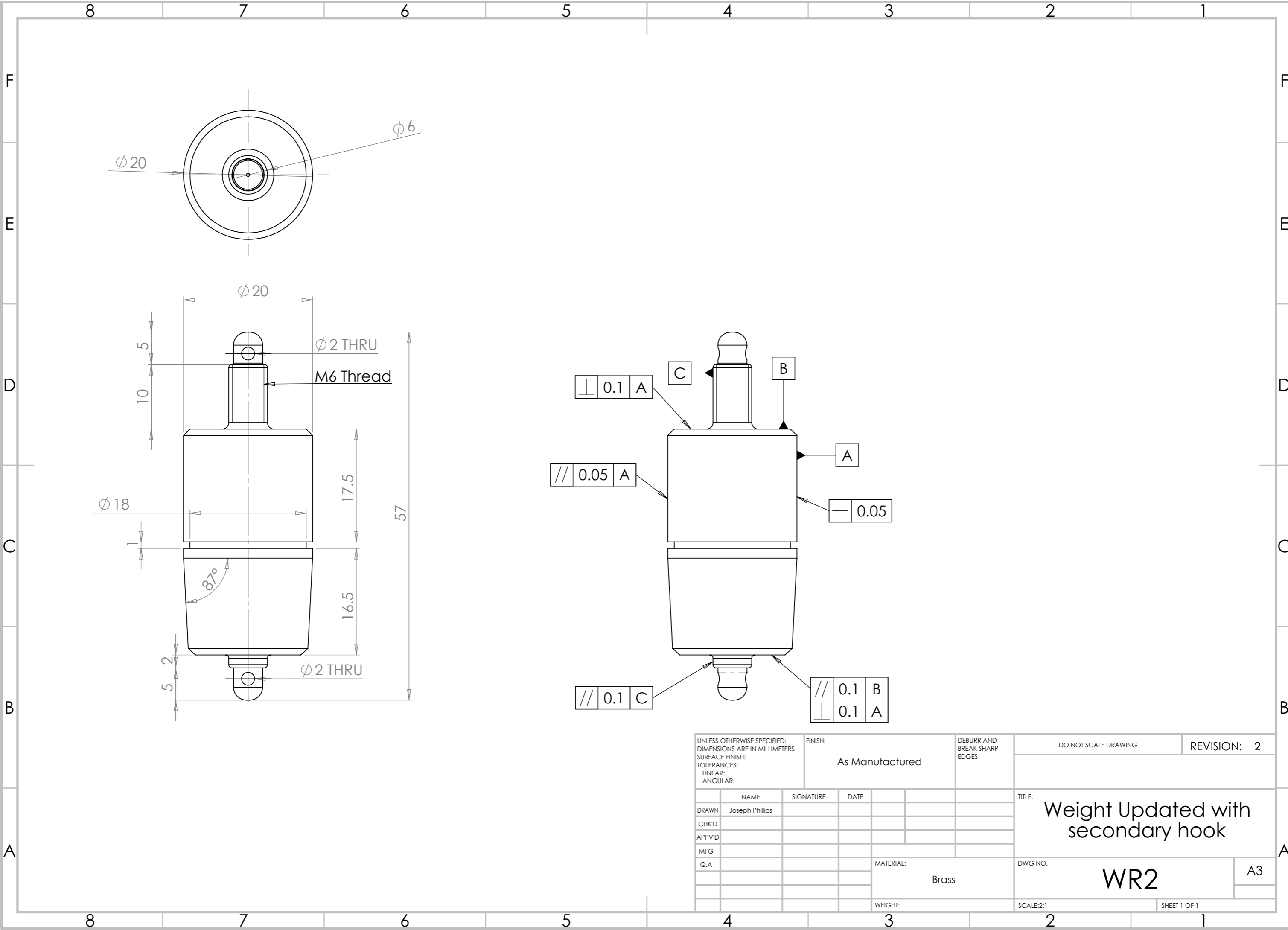

Supplement: Supplementary file 1 [file sensors-19-00936-s001.zip › Supplementary_data/Supplementary-data-1/Supplementary-data-1-weight-with-attachment.PDF]

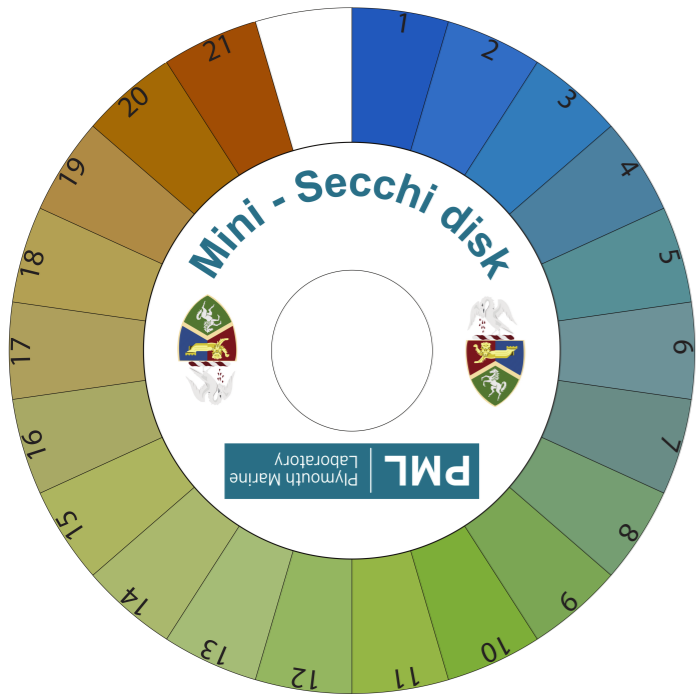

Supplement: Supplementary file 1 [file sensors-19-00936-s001.zip › Supplementary_data/Supplementary-data-4/Supplementary-data-4-vinyl-print-colour-scale-example.pdf]

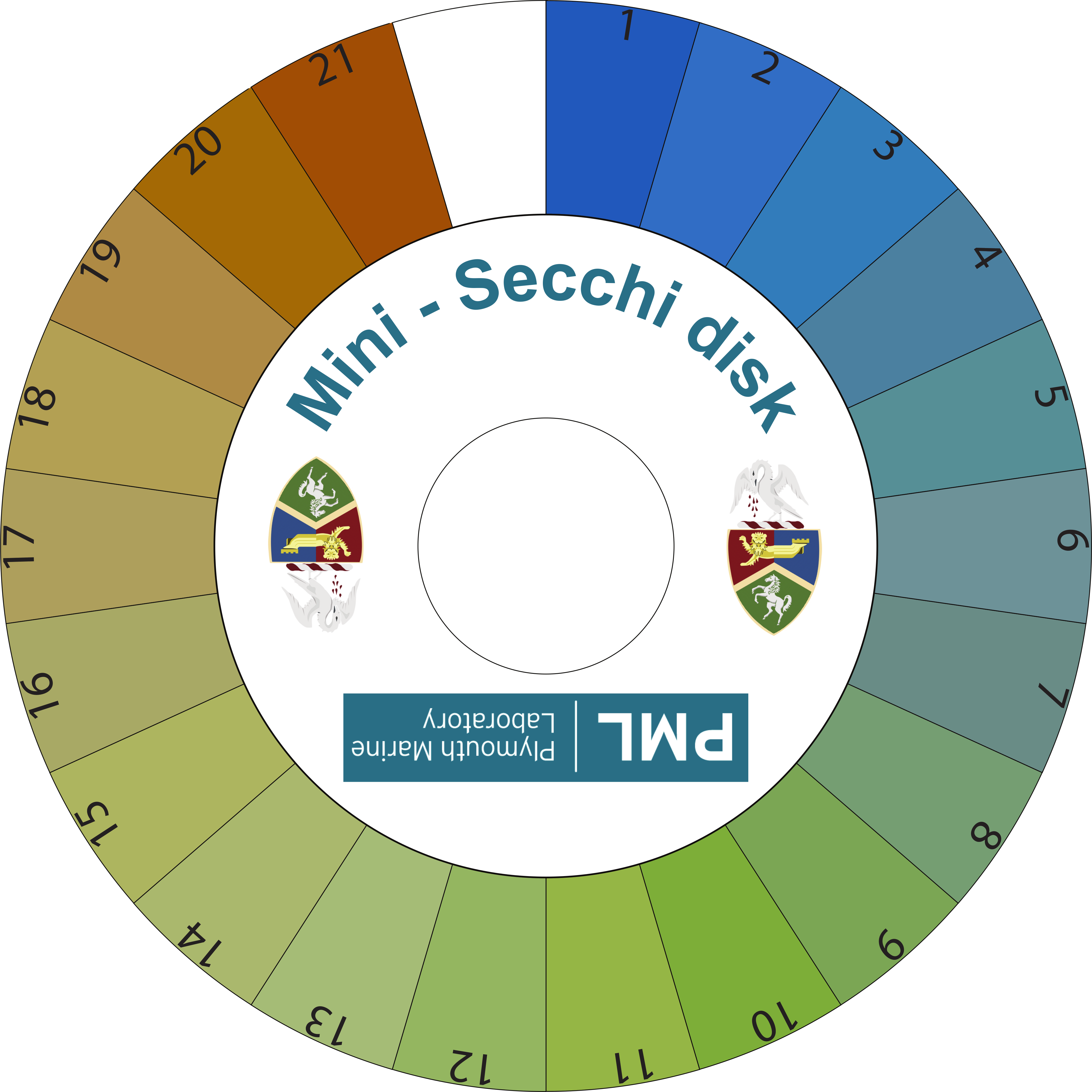

Supplement: Supplementary file 1 [file sensors-19-00936-s001.zip › Supplementary_data/Supplementary-data-4/Supplementary-data-4-vinyl-print-colour-scale-example.png]
